# Utilization of rehabilitation services among older persons with physical disabilities: an analysis of its association with socioeconomic status stratified by gender and age in China

**DOI:** 10.1186/s12889-026-27266-8

**Published:** 2026-04-07

**Authors:** Yi-Ran Wang, Lu Tan, Fan Zhang, Chen-Tao Zhang, Wan-Nian Liang

**Affiliations:** 1 Vanke School of Public Health, Tsinghua University, 30 Shuangqing Road, Beijing, 100084 China; 2 T.H. Chan School of Public Health, Harvard University, 677 Huntington Avenue, Boston, MA 02115 United States of America; 3 School of Media and Communication, Shanghai Jiao Tong University, 800 Dongchuan Road, Shanghai, 200240 China; 4 School of Ecology & Environment, Renmin University of China, 59 Zhongguancun Road, Beijing, 100872 China; 5 College of General Practice, Southern University of Science and Technology, 1088 Xueyuan Road, Shenzhen, 518000 China

**Keywords:** Older Persons, Disability, Socioeconomic Status, Rehabilitation Service Utilization, Association Mechanism

## Abstract

**Background:**

Rehabilitation is a critical method for preventing and controlling disabilities. However, the majority of the global demand for rehabilitation services remains unmet, particularly in low- and middle-income countries. This study aims to explore the complex and concealed stratified relationships between socioeconomic status and the utilization of rehabilitation services among older persons with physical disabilities (PD) from gender and age perspectives.

**Methods:**

A total of 19,782 observations of older persons with PD were included from the 2007–2013 China National Disabled Persons Condition Monitoring data. This study employs a multiplicative interaction effect model based on logistic regression to analyze the differential association between individual socioeconomic status and the utilization of rehabilitation services, considering gender and age.

**Results:**

From a gender perspective, the positive correlations between high income, high education and the utilization of rehabilitation services are smaller in males (high income: OR = 0.801, 95% CI 0.653–0.983; high education: OR = 0.679, 95% CI 0.433–1.066). From an age perspective, the positive correlation between high income and the utilization of rehabilitation services is greater among middle- to oldest-old individuals with PD (OR = 1.310, 95% CI 1.077–1.593).

**Conclusion:**

There are structural gender and age differences in the relationship between the socioeconomic status of older persons with PD and their utilization of rehabilitation services. It is recommended to enhance financial subsidies for rehabilitation services and implement tiered payment structures for low-income groups to alleviate their financial burden. To mitigate gender disparities in healthcare, efforts should focus on increasing awareness of rehabilitation services within health education programs, with community health workers playing a more active role in identifying and assisting those in need. Furthermore, strengthening support for family caregiving and integrating medical and nursing services within the framework of long-term care insurance are essential. Additionally, efforts should be made to the development of preventive rehabilitation programs and the establishment of a multi-tiered integrated rehabilitation system that ensures access to basic services for low-income populations and provides premium options to accommodate diverse needs.

## Background

Population aging is a global challenge, with significant implications for health systems, social care, and policy. International initiatives such as the World Health Organization’s (WHO) Decade of Healthy Ageing (2020–2030) emphasize the need for integrated and accessible health services for older persons, particularly in low- and middle-income countries. The United Nations Decade of Healthy Ageing (2021–2030) is well underway, with a central focus on measuring and monitoring changes in the functional ability and in the rights of older people worldwide to derive insights that can help develop and implement enabling environments and policies. These are very pertinent to the utilization of rehabilitation services.

Since the turn of the 21st century, the rapid aging of the population has exacerbated health challenges for older persons in China. The expected lifespan with disability among older persons is projected to increase from 5.78 years in 2015 to 7.44 years in 2030 and 11.45 years in 2050. Without effective preventive measures, the proportion of disabled older persons among the disabled population is expected to rise to 57% and 70% in 2030 and 2050, respectively [1]. Physical disabilities (PD) represent the largest category of disability in China, accounting for nearly 30% of the total disabled population [2]. Between 1987 and 2006, the prevalence of PD increased across different age groups in China, with the most significant increase observed among older persons [3].

Rehabilitation is a critical method for preventing and controlling disabilities. In 2008, the global burden of disease indicated that 92% of the disease burden could be alleviated through rehabilitation [4]. However, as highlighted by the WHO World Report on Disability (2011), the majority of the global demand for rehabilitation services remains unmet, particularly in low- and middle-income countries [5]. From the perspective of older persons in China, the second national sampling survey on disability in 2006 covered 354,859 older persons aged 60 and above, with 85,260 individuals suffering from disabilities. Of these, only 3,589 had utilized rehabilitation services, with a utilization rate of merely one-third [6]. The Chinese government has implemented various measures to promote the utilization of rehabilitation services. In 2010, the Ministry of Health in China issued a document named “Notice on the Inclusion of Additional Medical Rehabilitation Services in the Basic Medical Insurance Payment Scope”, mandating the inclusion of nine assessment and therapeutic rehabilitation projects nationwide. Since January 1, 2011, these nine rehabilitation projects have been funded proportionally by the basic medical insurance for urban employees, basic medical insurance for urban residents, and the new rural cooperative medical insurance for rural residents, which are the main types of health insurance in China. Overall, the reimbursement standards of these three types of insurance have some differences, but the gap is not significant. From an individual perspective, the utilization of health services is closely related to individual socioeconomic status [7]. While existing research predominantly focuses on the direct association between these two factors [8–11], the utilization of health services is also influenced by various factors such as gender [9, 12–15] and age [16–19]. There is relatively little research on the heterogeneous stratified relationship between individual socioeconomic status and health service utilization, especially concerning older disabled individuals. This study aims to explore the complex and concealed stratified relationships between socioeconomic status and the utilization of rehabilitation services among older persons with PD from gender and age perspectives. The primary focus is to explore the complex mechanisms behind the differentiated associations from economic, social, and cultural dimensions, structurally disaggregating the sources of effects of socioeconomic status differences, thereby identifying possible reasons and solutions for the inadequate utilization of rehabilitation services among the disabled in China. Our study contributes to this global discourse by providing insights into the structural disparities in rehabilitation services utilization among older persons in China. According to the explanation issued by the Office of the Second National Sampling Survey of Persons with Disabilities in China, physical disability refers to damage or dysfunction within the human locomotor system, resulting in limb deficiency, paralysis (paresis) of the limbs or trunk, deformities, and other related conditions. These impairments contribute to varying degrees of motor function loss and impose restrictions on daily activities and social participation. Physical disabilities include: the absence, deformity, or functional impairment of the upper or lower limbs due to injury, illness, or developmental abnormalities; deformities or functional impairments of the spine resulting from injury, illness, or developmental abnormalities; and functional impairments of the trunk or limbs caused by injuries, diseases, or developmental abnormalities of the central and peripheral nerves. Simple physical disability refers to the condition of having a physical disability without the coexistence of other types of disabilities (including visual disability, hearing disability, speech disability, intellectual disability, and mental disability).

## Methods

### Data

**Fig. 1:**
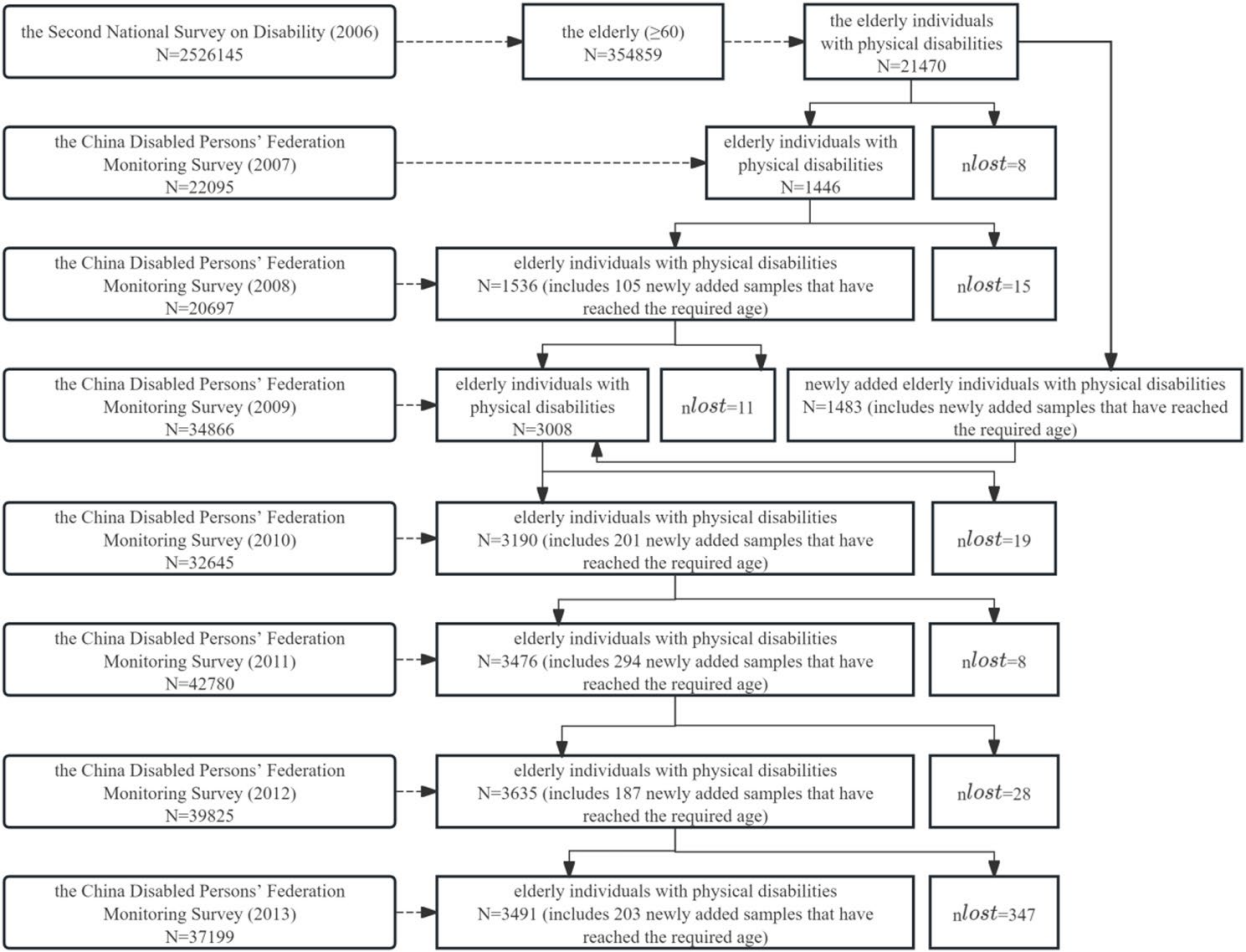
Sample size from 2007 to 2013

This study primarily uses data from the 2007–2013 China Disabled Persons Condition Monitoring Survey (hereinafter referred to as the monitoring survey), supplemented by the 2006 Second National Sample Survey on Disability (hereinafter referred to as the second survey). The monitoring survey served as a follow-up to the second survey [20]. The second survey aimed to grasp the quantity, structure, distribution, causes of disability, and rehabilitation, education, employment, and social participation of disabled persons in China during the survey year. This survey utilized stratified, multi-stage, cluster, probability-proportional-to-size sampling to obtain representative samples. A total of 734 counties (cities, districts), 2980 towns (townships, streets), and 5964 survey blocks were sampled, averaging around 420 people per block. The survey covered 771,797 households and 2,526,145 individuals, with a sampling ratio of 1.93‰ [21]. The second survey provided information on the disability grade, gender, urban-rural residence, and regional location (eastern, central, western) of disabled individuals. The monitoring survey randomly sampled disabled individuals from the second survey [22]. Specifically, the 2007 monitoring survey selected one survey block from each of the 734 county-level samples as the national monitoring sample unit and conducted a monitoring survey on all disabled individuals and their families within that block. The 2008 survey followed up on the 2007 samples, while the 2009 survey expanded the sample size from 734 to 1467 by adding one survey block in each county (city, district). Subsequent surveys in 2010, 2011, 2012, and 2013 followed up on the samples from the previous years. The monitoring survey covered the survival, development, and environmental conditions of disabled individuals, including their living conditions, rehabilitation, education, employment, community services, barrier-free environment, and legal services [7]. A total of 19,782 observations of older persons with PD were included from the 2007–2013 national disabled persons condition monitoring data, with the sample size over the years shown in Fig. 1.

### Measurement

The study targets older persons aged 60 and above with simple PD, referred to as “older persons with PD” hereafter. The hypothesis to be tested is that there are structural differences in the association between socioeconomic status and the utilization of rehabilitation services among older persons with PD based on gender and age. The variables are set as shown in Table 1.

#### Dependent variable

Utilization of rehabilitation services was selected as the outcome variable, which was defined as whether the individual used rehabilitation therapy, training, or assistive devices during the monitoring period.

#### Independent variables

Education level and annual per capita household income were selected as the independent variables representing the socioeconomic status of the individual. Education level were divided into no schooling, basic education (including primary and middle school), high school and above (including high school, technical secondary school, college and above). The total household income during the monitoring period (including annual wage income, urban business net income, rural business gross income, property income, transfer income, sale of assets income, and loan income) divided by household size to obtain annual per capita household income, divided into high, medium, and low tertiles based on annual income.

#### Moderating variables and controlling variables

Gender and age were set as moderating variables. In this study, we set older persons 60–69 years old as young-old individuals and 70 years old and above as middle- to oldest-old individuals. There were individual level control variables including disability grade, marital status and medical insurance coverage. Regional level control variables were also taken into consideration, including urban-rural residence, provincial per capita GDP, provincial average years of education, number of medical technicians per thousand people in the province (Table 1).

**Table 1:** Variable definitions

Variable	Definition
Dependent Variable	
Utilization of Rehabilitation Services	Whether the individual used rehabilitation therapy, training, or assistive devices during the monitoring period
Core Independent Variables	
Education Level	No schooling, basic education (including primary and middle school), high school and above (including high school, technical secondary school, college and above)
Annual Per Capita Household Income	The total household income during the monitoring period (the income sources include annual wage income, urban business net income, rural business gross income, property income, transfer income, sale of assets income, and loan income; pension is categorized as transfer income) divided by household size to obtain annual per capita household income, divided into high, medium, and low tertiles based on annual income
Individual Level Control Variables	
Disability Grade	Based on the disability grading standards from the 2006 s national sampling survey and the “Classification and Grading of Disabilities” (GB/T26341-2010), disabilities were categorized into four grades. Previous research [23] categorized grades 1–4 into mild disabilities (grade 4) and moderate to severe disabilities (grades 1–3)^a^
Gender	Male and female
Age	60–69 years old as young-old, 70 years old and above as middle- to oldest-old individuals
Marital Status	The actual marital status during the monitoring period, including never married, first marriage with spouse, remarriage with spouse, divorced, widowed. This study combines first marriage and remarriage with spouse into “with spouse” and other categories into “without spouse”
Medical Insurance Coverage	Whether the individual had basic medical insurance
Regional Level Control Variables	
Urban-Rural Residence	The residence status of the individual, categorized as urban or rural
Provincial Per Capita GDP	The per capita GDP of the province for the year following the monitoring period (e.g., 2007 for a monitoring period from April 1, 2006, to April 1, 2007), divided into high, medium, and low tertiles based on annual per capita GDP
Provincial Average Years of Education	The average years of education of the province for the year following the monitoring period (e.g., 2007 for a monitoring period from April 1, 2006, to April 1, 2007), divided into high, medium, and low tertiles based on annual average years of education
Number of Medical Technicians per Thousand People in the Province	The number of medical technicians per thousand people in the province for the year following the monitoring period (e.g., 2007 for a monitoring period from April 1, 2006, to April 1, 2007), divided into high, medium, and low tertiles based on annual data
Time Fixed Effects	Effects due to time variation

^a^According to the explanation issued by the Office of the Second National Sampling Survey of Persons with Disabilities in China, the screening, diagnosis, and assessment of persons with disabilities are primarily based on the degree of structural impairment and functional disability. Surveyors conduct household visits using the “Disability Screening Questionnaire for Individuals Aged 7 and Above” to identify suspected cases of disability. Those identified as potential cases are referred for further medical evaluation. Upon referral, doctors assess the patient’s limb deficiencies, standing and walking ability, as well as overall motor and functional capacity. The diagnosis is made based on a comprehensive physical examination, considering factors such as the location and severity of trunk, limb, and joint deformities, the extent and location of limb loss, muscle strength and tone, joint mobility, and the degree of limb functional impairment. Subsequently, based on the disability grading standards from the 2006 Second National Sampling Survey on Disability and the “Classification and Grading of Disabilities” (GB/T 26341-2010), disabilities were classified into four grades: level 1 (profound disability), level 2 (severe disability), level 3 (moderate disability), and level 4 (mild disability)

### Statistical analysis

Correlation analysis was conducted using a logistic regression model controlling for time fixed effects. For the differential association analysis, the predicted probabilities of each interaction group were first calculated when other variables except the main variables and interaction terms were set at their mean values. Subsequently, the differential correlations between individual socioeconomic status and the utilization of rehabilitation services were explored using the multiplicative interaction effect method based on the logistic regression model controlling for time fixed effects. The sources of effect differences were decomposed based on the relative size of the probabilities of rehabilitation service utilization in different interaction groups. Stata 16.0 software was used for the statistical analysis, with significance levels of 0.1, 0.05, and 0.01. The Variance Inflation Factor (VIF) was calculated for four models (including interactions between age and education, age and income, gender and education, and gender and income) to assess multicollinearity among the independent variables. The corresponding VIF values were 4.56, 3.35, 3.11, and 3.29, respectively, indicating that multicollinearity was not a significant issue in these models. Clustered standard errors were employed to address potential within-individual correlation over time that arises from repeated observations of the same individuals in different years.

## Results

### Gender and age differences in rehabilitation service utilization among older persons with PD of different socioeconomic status

#### Gender and age differences in rehabilitation service utilization among older persons with PD of different income levels

From the perspective of gender differences, overall, the rehabilitation service utilization rate among older persons with PD shows minimal differences between males and females (females slightly higher than males by less than 1% point). When disaggregated by income levels, the utilization rate for males is slightly higher than that for females in the low- and medium-income groups (by approximately 1–2% points). However, in the high-income group, females have a slightly higher utilization rate than males (by approximately 2% points), suggesting a tendency for higher-income females to utilize rehabilitation services. Both males and females exhibit the characteristic that higher income groups have correspondingly higher rehabilitation service utilization rates.

Regarding age differences, the overall rehabilitation service utilization rate for young-old (38.20%) is lower than that for middle- to oldest-old individuals (48.81%). This differentiated distribution is evident across different income groups. The utilization rate difference between young-old and middle- and oldest-old individuals with low, medium, and high per capita annual household income is approximately 6, 11, and 15% points, respectively, with the greatest difference seen in the highest income group. The gender and age differences in rehabilitation services utilization among older persons with PD of different income levels have been detailed in Table 2.

**Table 2:** Gender and age differences in rehabilitation service utilization among older persons with PD of different income levels

Variable	Category	Rehabilitation Service Utilization Rate (%)	Chi-square Test	Rehabilitation Service Utilization Rate by Income Level (%)		
				Low	Medium	High
Gender	Female	43.97	0.0020	37.53	39.80	54.37
	Male	43.94		38.70	42.13	51.09
Age	Young-old Individuals	38.20	224.3071***	33.95	35.85	44.62
	Middle- to Oldest-old Individuals	48.81		41.08	46.12	59.61

Asterisk indicates the statistical significance of the chi-square test (*** *p* < 0.01)

#### Gender and age differences in rehabilitation service utilization among older persons with PD of different education levels

In terms of gender differences, the overall rehabilitation service utilization rate is slightly higher for female older persons with PD (43.97%) compared to males (43.94%), indicating minimal gender differences. Disaggregating by education level, the slight difference in higher female utilization is mainly observed among those with no education and basic education. Among those with high school education and above, the gender gap widens significantly, with female utilization (65.20%) exceeding male utilization (54.92%) by approximately 11% points. This suggests a tendency for higher-educated females to utilize rehabilitation services more. Regardless of gender, both males and females show that higher education groups have correspondingly higher rehabilitation service utilization rates.

From the perspective of age differences, the overall utilization rate for middle- to oldest-old individuals is about 10% points higher than that for young-old. When broken down by age, both young-old and middle- to oldest-old individuals show the trend that higher education groups have higher rehabilitation service utilization rates. Among young-old, the utilization rate difference between those with no education and those with basic education is small (approximately 2% points). Among middle- to oldest-old individuals, the corresponding difference increases to 5% points, and across all age groups, those with high school education and above show significantly higher utilization rates than those with no education or basic education. The gender and age differences in rehabilitation service utilization among older persons with PD of different education levels have been detailed in Table 3.

**Table 3:** Gender and age differences in rehabilitation service utilization among older persons with PD of different education levels

Variable	Category	Rehabilitation Service Utilization Rate (%)	Chi-square Test	Rehabilitation Service Utilization Rate by Education Level (%)		
				No Education	Basic Education	High School and Above
Gender	Female	43.97	0.0020	42.61	43.70	65.20
	Male	43.94		41.60	43.30	54.92
Age	Young-old Individuals	38.20	224.3071***	35.80	37.81	51.09
	Middle- to Oldest-old Individuals	48.81		45.29	50.62	65.08

Asterisk indicates the statistical significance of the chi-square test (*** *p* < 0.01)

In summary, older persons with PD who have higher socioeconomic status have correspondingly higher rehabilitation service utilization rates. This trend is consistent across different gender and age groups, though certain differences remain. Specifically, female and middle- to oldest-old individuals with high school education and above or high per capita annual household income exhibit significantly higher utilization rates compared to other groups. Hence, subsequent analyses in this study will explore the differential association between socioeconomic status and rehabilitation service utilization among older persons with PD, disaggregated by gender and age.

### Gender differences in the correlation between socioeconomic status and rehabilitation services utilization among older persons with PD

#### Gender differences in the correlation between income level and rehabilitation services utilization among older persons with PD

**Fig. 2:**
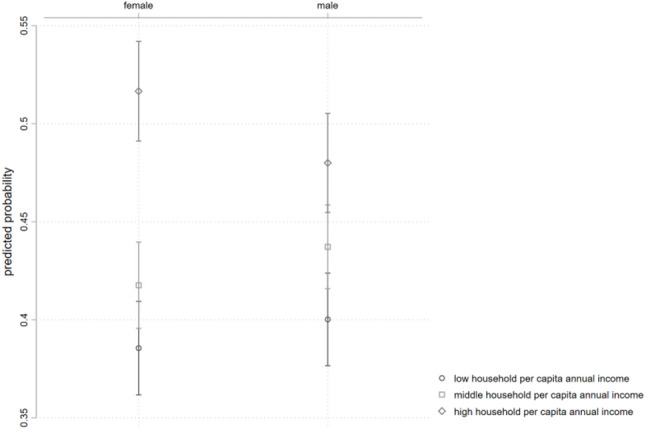
Predicted probability of rehabilitation service utilization by gender and income level

Figure 2 illustrates the predicted probability of rehabilitation services utilization for different interaction groups based on gender and income level. Compared to older male adults with PD, females in the high-income group have a relatively higher probability of utilizing rehabilitation services, while the utilization probability in the low- and medium-income groups is relatively lower.

Table 4 shows the correlation between per capita annual household income and rehabilitation services utilization among older persons with PD across different gender groups, with females as the reference group. The results indicate that, compared to the low-income reference group, the correlation of high income and rehabilitation services utilization for older male adults with PD is significantly lower than that for females (OR = 0.801, 95% CI 0.653 ~ 0.983). This suggests that high-income males are about 20% less likely to utilize rehabilitation services compared to high-income females.

**Table 4:** Gender differences in the correlation between per capita annual household income and rehabilitation services utilization among older persons with PD

Interaction Term	OR	S.E.	z	95% CI
Gender × Per Capita Annual Household Income (reference group: female, low income)				
Male × Medium Income	1.020	0.092	0.22	0.855 ~ 1.218
Male × High Income	0.801**	0.084	-2.12	0.653 ~ 0.983

OR = odds ratio; S.E. = standard error. Asterisk indicates statistical significance of the estimated odds ratio (** *p* < 0.05)

#### Gender differences in the correlation between education level and rehabilitation services utilization among older persons with PD

**Fig. 3:**
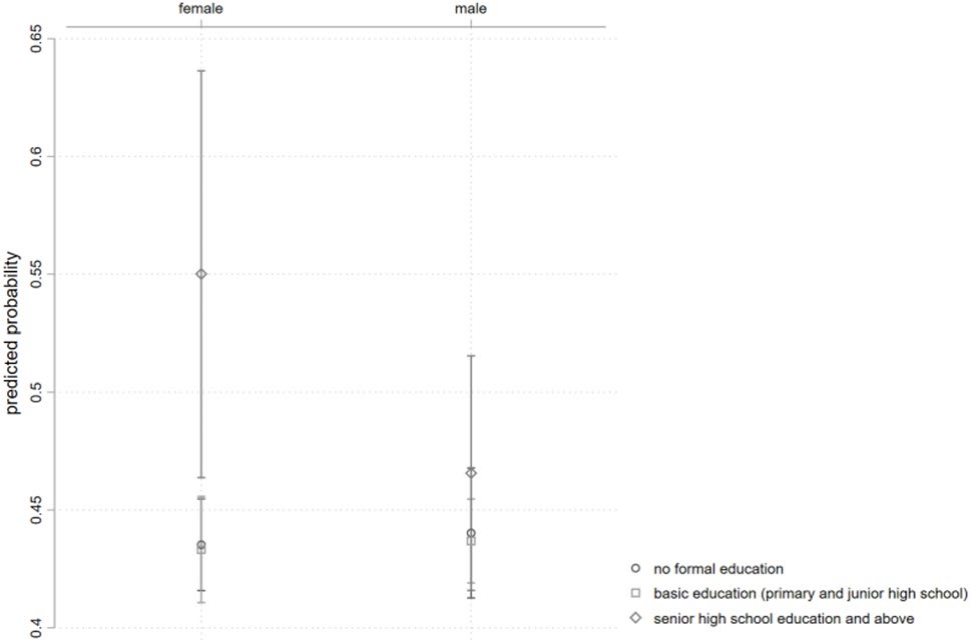
Predicted probability of rehabilitation service utilization by gender and education level

Figure 3 illustrates the predicted probability of rehabilitation service utilization for different interaction groups based on gender and education level. Females with higher education levels have a relatively higher probability of utilizing rehabilitation services compared to males. The gap in utilization probability between genders is smaller in the low and medium education groups.

Table 5 displays the gender differences in the correlation between education level and rehabilitation services utilization among older persons with PD, with females as the reference group. The results show that, compared to the no education reference group, the correlation of high school and above education and rehabilitation services utilization for older male adults with PD is significantly lower than that for females (OR = 0.679, 95% CI 0.433 ~ 1.066), indicating that highly educated males are about 32% less likely to utilize rehabilitation services compared to highly educated females.

**Table 5:** Gender differences in the correlation of education level and rehabilitation services utilization among older persons with PD

Interaction Term	OR	S.E.	z	95% CI
Gender × Education Level (reference group: female, no education)				
Male × Basic Education	0.994	0.093	-0.06	0.827 ~ 1.195
Male × High School and Above	0.679*	0.156	-1.68	0.433 ~ 1.066

OR = odds ratio; S.E. = standard error. Asterisk indicates statistical significance of the estimated odds ratio (* *p* < 0.1)

### Age differences in the correlation of socioeconomic status and rehabilitation services utilization among older persons with PD

#### Age differences in the correlation of income level and rehabilitation services utilization among older persons with PD

**Fig. 4:**
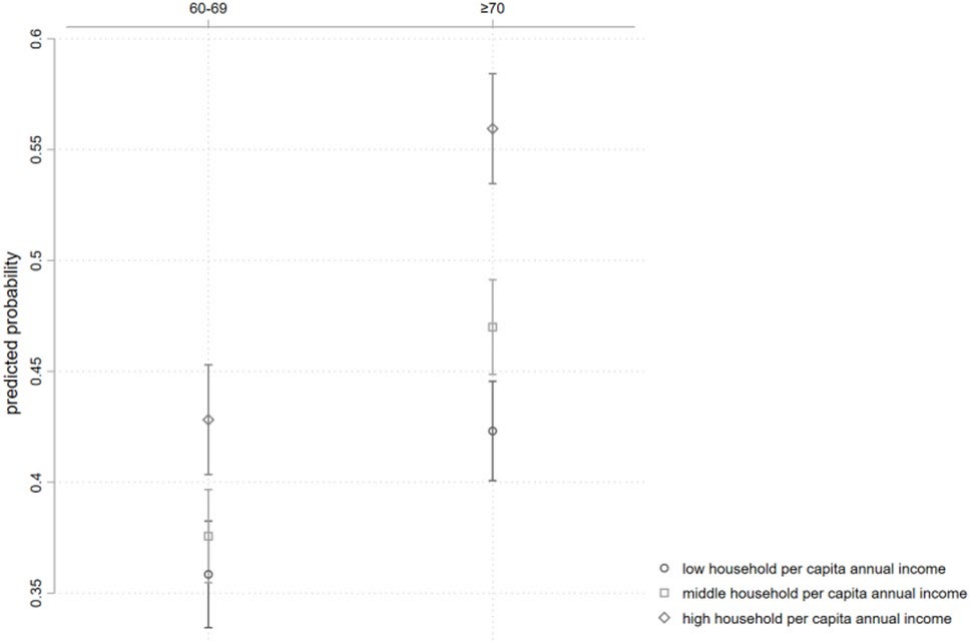
Predicted probability of rehabilitation services utilization by age and income level

Figure 4 illustrates the predicted probability of rehabilitation service utilization for different interaction groups based on age and income level. Compared to young-old with PD, middle- to oldest-old individuals show relatively higher probabilities of utilizing rehabilitation services across all income levels.

Table 6 illustrates the correlation of per capita annual household income and the utilization of rehabilitation services among older persons with PD in China, across different age groups, with young-old serving as the reference group. The results indicate that, with low per capita annual household income as the within-group reference, the correlation between medium per capita annual household income and the utilization of rehabilitation services by middle- to oldest-old individuals with PD is not significantly different from the correlation in young-old individuals. However, the correlation between high per capita annual household income and the utilization of rehabilitation services by middle- to oldest-old individuals is significantly higher than the correlation in young-old individuals (OR = 1.310, 95% CI 1.077 ~ 1.593). This suggests that middle- to oldest-old individuals with high income are about 31% more likely to utilize rehabilitation services compared to young-old individuals with high income.

**Table 6:** Age differences in the correlation of per capita annual household income and rehabilitation services utilization among older persons with PD

Interaction Term	OR	S.E.	z	95% CI
Age × Per Capita Annual Household Income (Reference group: young-old; within-group reference: low per capita annual household income)				
Middle- to oldest-old individuals × Medium income	1.130	0.099	1.39	0.951 ~ 1.342
Middle- to oldest-old individuals × High income	1.310***	0.131	2.71	1.077 ~ 1.593

*OR* odds ratio, *SE* standard error

Asterisk indicates statistical significance of the estimated odds ratio (*** *p* < 0.01)

#### Age differences in the correlation between educational level and rehabilitation services utilization among older persons with PD

**Fig. 5:**
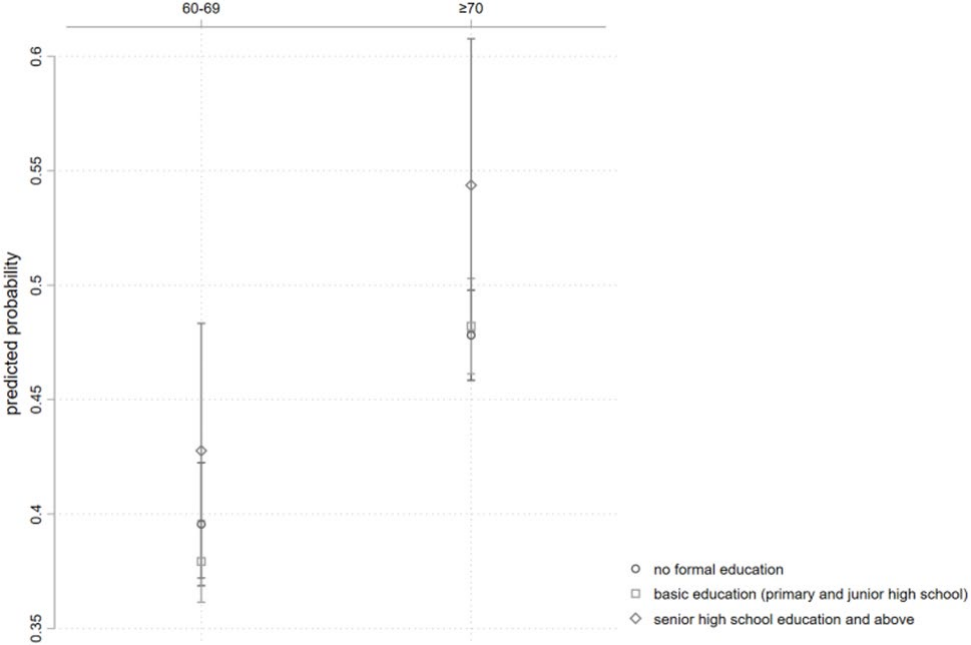
Predicted probability of rehabilitation service utilization by age and educational level

Figure 5 illustrates the predicted probability of rehabilitation service utilization for different interaction groups based on age and educational level. Compared to young-old individuals with PD, middle- to oldest-old individuals show relatively higher probabilities of utilizing rehabilitation services across all educational levels.

Table 7 illustrates the correlation between educational level on the utilization of rehabilitation services among older persons with PD in China, across different age groups, with young-old individuals serving as the reference group. The results indicate that, with no schooling as the within-group reference, the correlation between basic education and high school or above education and the utilization of rehabilitation services by middle- to oldest-old individuals with PD is not significantly different from the correlation in young-old individuals.

**Table 7:** Age Differences in the Correlation between Educational Level and Rehabilitation Services Utilization Among Older Persons with PD

Interaction Term	OR	S.E.	z	95% CI
Age × Educational Level (Reference group: young-old individuals; within-group reference: no schooling)				
Middle- to oldest-old individuals × Basic education	1.094	0.097	1.01	0.919 ~ 1.302
Middle- to oldest-old individuals × High school and above education	1.149	0.219	0.73	0.791 ~ 1.668

*OR* odds ratio, *SE* standard error

## Discussion

### Gender differences in the correlation between socioeconomic status on rehabilitation service utilization among older persons with PD

The research findings indicate that, in terms of income level, the positive correlation between high income and the utilization of rehabilitation services by older male adults with PD is less than that for their female counterparts (Effect 1). Regarding educational level, the positive correlation between higher education (high school and above) and the utilization of rehabilitation services by older male adults with PD is also less than that for their female counterparts (Effect 2). This could be attributed to the convergence of family resources and the gender reversal in educational attainment mitigating the traditional gender norms favoring males in the allocation of rehabilitation resources.

Analyzing the probability distribution, Effect 1 arises partly from the lower probability of low-income older female adults with PD utilizing rehabilitation services compared to their male counterparts, and partly from the higher probability of high-income older female adults utilizing these services compared to high-income older male adults. Effect 2 primarily stems from the higher probability of highly educated older female adults utilizing rehabilitation services compared to highly educated men, with a slight influence from the lower probability of less educated women utilizing these services compared to less educated men.

Feminist economics posits that women’s greater susceptibility to relative poverty is a long-standing, global issue [24]. In the 1970s, American scholar Valentine M. Mogerharden discovered that apart from children, the most severely affected by poverty were female-headed households and low-income women, initiating conceptual discussions around the “feminization of poverty” [25]. Merton’s cumulative advantage theory emphasizes that with age, an individual’s advantages and disadvantages accumulate, exacerbating gender inequality through the “Matthew effect” [26]. From this perspective, the dual vulnerabilities of women and aging exacerbate their disadvantaged position in utilizing rehabilitation services. The “feminization of poverty” as a global challenge manifests regionally; in China, it is rooted in “inequitable intra-household resource allocation” [24, 27]. Particularly in rural areas, gender inequality in health resource distribution is pronounced [28]. According to UN Women (2019), despite improvements in women’s rights over the past decades, gender inequality within households remains pervasive. This is attributed to the prolonged patriarchal societal structure and cultural consciousness, which have ingrained traditional gender norms characterized by female dependency [29]. Consequently, institutional designs and policy care aimed at emancipating women’s productivity and improving their status have yet to structurally reconstruct the roles and status of women, especially those from impoverished families [24]. Women’s autonomy in family decision-making and resource allocation remains significantly lower than men’s [30], particularly in economically disadvantaged families. From an educational perspective, less educated women lack cognitive autonomy and decision-making independence, making them more likely than men to be resource-deprived under the influence of “inequitable intra-household resource allocation.”

In contrast, older female adults with PD who are high-income and well-educated exhibit utilization probabilities for rehabilitation services that are opposite to those of disadvantaged female groups. From an income perspective, affluent families possess relatively ample resources, mitigating the adverse effects of “inequitable intra-household resource allocation” on women’s disadvantaged status. Educationally, social transformations have significantly challenged traditional patriarchal gender norms [31]. In the establishment of new gender norms characterized by equality, highly educated women, with their emancipated thinking and more independent family and social status, have become pioneers in breaking traditional constraints. The “WHO World Health Statistics 2019” report indicates that women have higher life expectancy and healthy life expectancy than men: men’s average life expectancy is 69.8 years, shorter by 4.4 years compared to women (74.2 years), with women having longer healthy life expectancy at birth and at age 60. WHO attributes these gender differences in health primarily to differences in health literacy: in areas where women struggle to access healthcare, the life expectancy gap between genders is minimal; in regions where men and women face the same diseases, men tend to seek medical care less frequently than women [32]. This suggests that compared to men, women have higher health sensitivity [12, 13, 14], with a tendency towards health behaviors. Within the overall suppression of female access to health resources by traditional gender norms, high-income, well-educated women, through their independent ability to access health resources, are “liberated” from this suppression. Thus, female health literacy and equal status in health resource allocation are gradually achieved through the interplay of “overall suppression by traditional gender norms” and “enhancement of women’s independent thinking, decision-making, and resource acquisition abilities.” Affluent women, especially those with excellent educational backgrounds, continuously break through patriarchal constraints, unleashing their inherent health-oriented tendencies.

Furthermore, from a psychological perspective, as individuals age, personality traits change. The Kansas City study, a pioneering longitudinal study on middle-aged and older elderly personality, found that with age, older female adults exhibit more assertive and self-centered traits [33]. For men, traditional patriarchal gender norms foster masculine and resilient traits [29], which high socioeconomic status may further reinforce, leading older male adults to reject the vulnerability associated with disability and resist the “low posture” behavior and “weak” psychological state implied by utilizing rehabilitation services.

### Age differences in the correlation between socioeconomic status on rehabilitation service utilization by older persons with PD

The research findings indicate that, in terms of income level, the positive effect of high income on the utilization of rehabilitation services by middle- to oldest-old individuals with PD is significantly greater than that for young-old individuals with PD (Effect 3). This may result from the “high cost-low benefit” dilemma in health investment for disabled individuals and the traditional concept of “not wanting to burden children,” which inhibits rehabilitation service utilization among middle- to oldest-old individuals from low-income families. In contrast, health investments by individuals from high-income families, with minimal impact on the quality of life of other family members, promote the utilization of rehabilitation services among middle- to oldest-old individuals.

The probability distribution of rehabilitation service utilization across different income levels and age groups shows that Effect 3 primarily results from a larger increase in utilization probability for high-income older persons as they age, compared to a smaller increase for low-income older persons.

Aging is accompanied by a decline in bodily functions [34], and the immune system’s ability to effectively resist and repair the body’s weakened state diminishes with age [35]. Consequently, compared to young-old individuals with PD, middle- to oldest-old individuals have a higher demand for health interventions, leading to a greater likelihood of utilizing rehabilitation services. The age effect theory posits that as individuals age, especially in the old age, the impact of socioeconomic status on individual health gradually diminishes, and health levels among older persons with different socioeconomic statuses tend to converge, reducing health inequality [26]. Based on the age effect theory, in the context of converging health levels among middle- to oldest-old individuals with PD of different socioeconomic statuses, the influence of socioeconomic status on their utilization of rehabilitation services may become more pronounced. The interaction between age and income level shows significant differences in the correlation between high income and the likelihood of utilizing rehabilitation services among older persons with PD across different age groups. Compared to young-old individuals, the positive effect of high income on the utilization of rehabilitation services by middle- to oldest-old individuals is more pronounced.

From an economic perspective, the utilization of rehabilitation services by older persons is essentially a health investment. However, the relatively severe health condition of middle- to oldest-old individuals with PD can lead to a “high cost-low benefit” dilemma in health investment. Additionally, compared to young-old individuals, the “elderly dividend” (individual labor’s contribution to family and social development) gradually diminishes. Thus, considering these factors, families of middle- to oldest-old individuals with low income may be more reluctant to bear the high sunk costs due to limited economic capacity. In contrast, affluent families are less constrained by economic capacity, and health investments to maintain the quality of life for older persons have a lesser impact on the overall quality of life of family members.

From a cultural perspective, the Chinese belief that “longevity is a blessing” is more likely to be rooted in affluent families. As the ancient text “Records of the Grand Historian” states, “When the granaries are full, the people know propriety and moderation.” However, in economically disadvantaged families, the high cost, long duration, and low efficiency of disability rehabilitation can easily lead to catastrophic health expenditures. Consequently, the belief that “longevity is a blessing” in affluent families becomes a major risk factor for impoverishment or return to poverty in economically disadvantaged families. Additionally, the “social selective positivity” might suppress rehabilitation service utilization behavior among middle- to oldest-old individuals with PD in impoverished families. When they perceive themselves as nearing the end of their lives, they might prefer choices that bring positive emotions, such as “not adding to their children’s burden,” which is a common positive emotional choice among frail older persons in impoverished families [36].

### Limitations

In the core explanatory variables of this study, there are individual-level non-time-varying variables (educational level). Therefore, a two-way fixed effects model controlling for time and individual fixed effects could not be used, and logistic regression analysis with time fixed effects and provincial control variables was employed instead. (2) There have been some changes in China’s health policy framework in recent years. Due to the lack of updated national data, this study cannot directly assess the specific impacts of these policy adjustments on the utilization of rehabilitation services among the disabled older adults. However, the impact mechanisms of SES on the utilization of rehabilitation services revealed in this study exhibit strong structural integrity and long-term stability, and the underlying mechanisms of gender and age differences (such as traditional gender norms and health investment attitudes) remain persistent. Therefore, this study retains policy and academic value in the current context. (3) Due to data constraints, we were unable to directly measure individual-level distances to medical facilities or geographic accessibility.

## Conclusions

There are structural gender and age differences in the relationship between the socioeconomic status of older persons with PD and their utilization of rehabilitation services, influenced by social, economic, and cultural factors. The positive correlations between high income, high education and the utilization of rehabilitation services are weaker in males with PD, and the positive correlation between high income and the utilization of rehabilitation services is stronger among middle- to oldest-old individuals with PD. It is recommended to enhance financial subsidies for rehabilitation services and implement tiered payment structures for low-income groups to alleviate their financial burden. To mitigate gender disparities in healthcare, efforts should focus on increasing awareness of rehabilitation services within health education programs, with community health workers playing a more active role in identifying and assisting those in need. Furthermore, strengthening support for family caregiving and integrating medical and nursing services within the framework of long-term care insurance are essential. Additionally, efforts should be made to the development of preventive rehabilitation programs and the establishment of a multi-tiered integrated rehabilitation system that ensures access to basic services for low-income populations and provides premium options to accommodate diverse needs.

## Data Availability

The data that support the findings of this study are available from the Institute of Population Research of Peking University (IPR). Restrictions apply to the availability of these data, which were used under license for this study. Data are available with the permission of IPR. Requests to access these datasets should be directed to 00-86-010-62751974.
